# High-Adhesive Flexible Electrodes and Their Manufacture: A Review

**DOI:** 10.3390/mi12121505

**Published:** 2021-11-30

**Authors:** Yingying Xiao, Mengzhu Wang, Ye Li, Zhicheng Sun, Zilong Liu, Liang He, Ruping Liu

**Affiliations:** 1 Beijing Engineering Research Center of Printed Electronics, Beijing Institute of Graphic Communication, Beijing 102600, China; xiaoyingying0420@gmail.com (Y.X.); wangmengzhu777@gmail.com (M.W.); liye@bigc.edu.cn (Y.L.); sunzhicheng@bigc.edu.cn (Z.S.); 2 Division of Optics, National Institute of Metrology, Beijing 100029, China; liuzl@nim.ac.cn; 3 School of Mechanical Engineering, Sichuan University, Chengdu 610065, China; hel20@scu.edu.cn

**Keywords:** electrical physiology, electrode, flexible electronics, high-adhesive, manufacturing, printed electronic

## Abstract

All human activity is associated with the generation of electrical signals. These signals are collectively referred to as electrical physiology (EP) signals (e.g., electrocardiogram, electroencephalogram, electromyography, electrooculography, etc.), which can be recorded by electrodes. EP electrodes are not only widely used in the study of primary diseases and clinical practice, but also have potential applications in wearable electronics, human–computer interface, and intelligent robots. Various technologies are required to achieve such goals. Among these technologies, adhesion and stretchable electrode technology is a key component for rapid development of high-performance sensors. In last decade, remarkable efforts have been made in the development of flexible and high-adhesive EP recording systems and preparation technologies. Regarding these advancements, this review outlines the design strategies and related materials for flexible and adhesive EP electrodes, and briefly summarizes their related manufacturing techniques.

## 1. Introduction

With continuous advancement in medical technology, some medical conditions, such as epilepsy, congenital heart defects, arrhythmia, and glaucoma, are moving from treatment to prevention increasingly. One example of remote health monitoring is the electrical physiology (EP) monitoring, which can increase the success rate of treatment and reduce healthcare expenses [1]. Therefore, communicating and acquiring EP signals has become the biggest challenge to this field. As a method for measuring electrical signals, electrodes are the most critical technology in the EP working system. EP signal recording applications include the brain–computer interface, human–computer interface (HCI) [2,3,4], and human–machine interface (HMI) [5], etc., in addition to some innovative applications, such as attention detection [6] and the development of a point-of-care (POC) [7] diagnostic platform.

An EP signal is the membrane potential caused when the ion composition of cell interior is substantially different from the milieu outside of cell when excitable cells (including nerve cells, cardiomyocytes, muscle cells, etc.) undergo electrochemical activities [8]. Excitable cells can generate action potentials when stimulated while non-excitable cells only have resting potentials and do not generate action potentials. The complex electrical changes in the brain, heart, and other organs are the sum of the changes in the electrochemical activities of the cells of which they are made up.

For capture and analysis of EP signals, electrodes are essential components, which are composed of base, interface, and packaging materials. According to the different ways of detecting EP signals, electrodes are divided into two types: non-implantable (external) and implantable (internal). Non-implantable electrodes are used for body surface detection [9,10] and are not harmful to the human body. The surface of human skin is divided into epidermis and dermis, and the stratum corneum on the epidermis has excellent impedance characteristics [11]. Therefore, it is necessary to overcome the influence of the stratum corneum as much as possible when using non-implantable electrodes for EP signal detection. At present, most non-implantable electrodes used in medical monitoring systems are commercial silver/silver chloride (Ag/AgCl) wet electrodes [12,13,14,15], which have been used for a long time [16,17]. Because the epidermal layer on the surface of the human skin is insulated (high impedance), the Ag/AgCl wet electrode needs conductive gel as the electrolyte channel to reduce the electrode-skin interface impedance (EII) between the skin and AgCl. An adhesive is also necessary to prevent the electrode from falling off the skin. Thus, the Ag/AgCl electrode detects a good signal EP. However, because the liquid in the gel is easily volatilized and its tensile strength is easily reduced, the Ag/AgCl wet electrode is prone to signal degradation during long-term continuous monitoring. This results in the electrodes needing to be replaced every day while it may also cause skin allergies [1,18]. To solve these problems, researchers have studied dry electrodes that do not require skin preparation [19].

The traditional Ag/AgCl wet electrode has the abovementioned problems because it cannot make conformal contact with the skin and thus cannot form a stable contact interface. In addition, since the electrode itself is not adhesive, it easily falls off the skin. Therefore, research and preparation of high-adhesive flexible EP electrodes is underway. Additionally, the application range of high-adhesion flexible EP electrodes is related to electrode function and characteristics and is related to its manufacturing cost. The traditional manufacturing process based on a micro-electro-mechanical system has limited application in the field of EP electrodes due to its high price and low efficiency. The continuous development of printed electronics technology and large-scale roll-to-roll (R2R) manufacturing will help achieve high-precision mass manufacturing of highly adherent flexible EP electrodes [9,20]. To develop high-adhesive flexible EP electrodes can produce real-time monitoring effects in disease detection, helping more patients to reduce the risk of sudden accidents. Toward human–computer interaction and intelligent manufacturing, it can bring unlimited possibilities for human life. There are several review articles about the non-implanted electrode area [21,22,23] and most of them are from the material or structure point of view.

**Figure 1:**
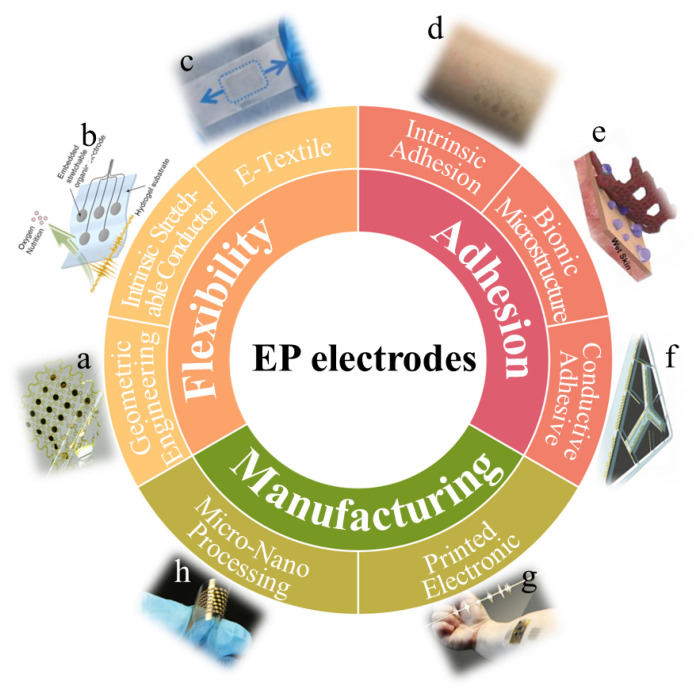
A summary of research on high-adhesive flexible bioelectrodes and their manufacturing methods. (**a**) Schematic diagram of an electrode of the island bridge structure of geometric engineering. Reproduced with permission from [24]. Copyright 2016, American Chemical Society. (**b**) Concept and advantages of a hydrogel-based organic subdural electrode. Reproduced with permission from [25]. Copyright 2019, The Authors. (**c**) Photograph of graphene fabric electrode. Reproduced with permission from [26]. Copyright 2020, American Chemical Society. (**d**) Photograph of breathable tattoo electrodes. Reproduced with permission from [27]. Copyright 2017, American Chemical Society. (**e**) Photograph of an electrode patch inspired by octopus structure. Reproduced with permission from [28]. Copyright 2020, American Chemical Society. (**f**) Photograph of a conductive adhesive coated on electroencephalogram electrodes. Reproduced with permission from [29]. Copyright 2014, The Authors. (**g**) Stretchable sensors from contactless direct printing. Reproduced with permission from [30]. Copyright 2020, American Chemical Society. (**h**) Micro-nano processing to prepare electromyography electrodes. Reproduced with permission from [31]. Copyright 2018, The Authors.

This review focuses on the recent progresses in non-implantable skin electrodes, with particular interests in implementation strategies for electrode flexibility, adhesion, and manufacturing technology Figure 1. We refer non-implantable skin electrodes that record EP signals simply as “EP electrodes”. We first introduce the three preparation strategies of flexible EP electrodes (geometric engineering, intrinsic stretchable conductor, and e-textile), which is then followed by research updates achieved so far toward those features. In particular, strategies to enhance adhesion (intrinsic adhesion, bionic microstructure, and conductive adhesive) based on the preparation of flexible EP electrodes is discussed. Furthermore, we also introduce and compare subtractive and additive manufacturing in the preparation of EP electrodes. A summary of the progress, perspective challenges and opportunities regarding EP electrodes is elaborated.

## 2. Fabrication Strategies for Flexible and Stretchable EP Electrodes

The ideal characteristics of flexible EP electrodes are high stretchability and excellent electrical conductivity. Human skin can survive a mechanical strain of ≈15% without irreversible damage [32] and flexible EP electrodes are required to possess mechanical performance matched with human skin [33]. In order to ensure conformal contact between electrode and skin, and prevent premature failure and loss of function [34], the electrodes are required to have a certain degree of bending and stretching properties. With the development of micromachining technology and nanoparticles, researchers used flexible materials with low Young’s modulus as substrates for electrode preparation. Therefore, it is necessary to design an integrated and inherently stretchable device. The preparation strategies of three stretchable EP electrodes will be introduced later, and the corresponding detailed discussion will be carried out from the perspective of materials.

### 2.1. Geometric Engineering Fabrication

The geometric engineering fabrication method does not change the characteristics of the material itself, but only the geometric pattern design of rigid materials to obtain particular tensile properties [35]. Generally speaking, conventional conductive rigid materials (such as Si, Au, Pt, etc.) are used in the design of a microstructure of geometric patterns to obtain stretchability. Meanwhile, organic materials with stretchable properties are selected as the substrate, supporting rigid body materials. For example, the rigid island structure in traditional flexible devices transfers stress from the rigid components of the device to the surrounding stretchable conductive connecting wires. Each rigid island can be regarded as a unit or contact point of the device. The rigid islands are connected by micro-structured conductive interconnects to obtain a quantitative bending strain [24,36].

**Figure 2:**
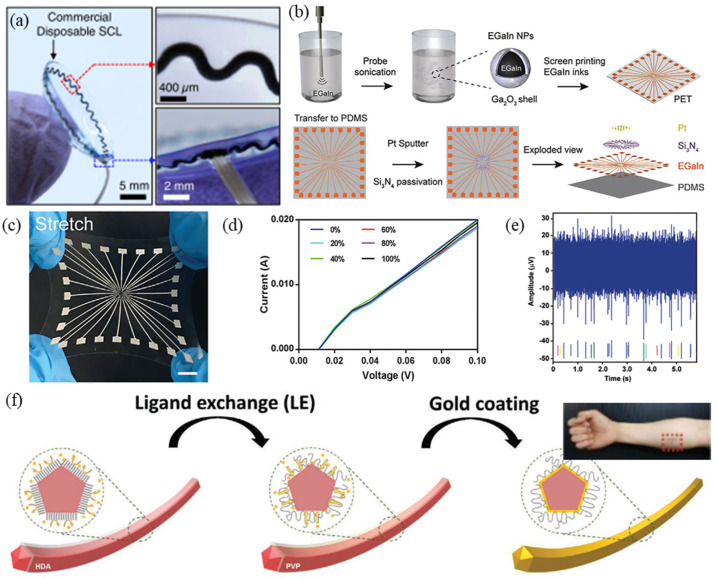
Summary of preparation strategies for flexible electrical physiology electrodes. (**a**) Photographs of a corneal sensor with inset images highlighting the serpentine layout and its seamless integration with the connection wire. Reproduced with permission from [38]. Copyright 2021, The Authors. (**b**) Schematic illustration of the fabrication of a stretchable electrode array (SEA). (**c**) Snapshots of a SEA with high flexibility and stretchability. (**d**) I–V curves of the conductive trace with different strains. (**e**) In vitro neural signal recording by the SEA (1 Hz to 3 kHz; noise floor: −20 µV). (**b**–**e**) are reproduced with permission from [41]. Copyright 2021, John Wiley and Sons. (**f**) Schematic of the gold deposition process for an electrical physiology (EP) electrode. Reproduced with permission from [42] Copyright 2020, John Wiley and Sons.

Geometric engineering is becoming ever more common and able to obtain higher plasticity. For example, Shahandashti et al. used polyimide (PI) film with low creep performance and high tensile strength as the support material for a Au/Cu double layer (6 μm/35 μm)[37]. The PI was placed on a glass substrate and a photolithography process was used to construct a 200-µm-wide spring-shaped interconnection line and electrical pads; finally, it was pasted on polydimethylsiloxane (PDMS) to complete the electrode production. Experiments showed that the electrode can withstand 25% tensile deformation and the electrode impedance is equivalent to that of a standard Ag/AgCl wet electrode, showing good performance in electroencephalogram (ECG) and electromyography (EMG) signal detection [37]. Kim et al. printed a snake-shaped elastomer ink on a glass substrate coated by a water-soluble polyvinyl alcohol (PVA) layer. The elastomer ink was composed of silver flake-filled polystyrene-b-poly(ethylene-co-butylene)-b-polystyrene (AgSEBS) and polydimethylsiloxane (PDMS); after removing the PVA layer, gold was plated on the AgSEBS to improve conductivity, and finally, the ink was transferred to disposable contact lenses and introduced conductive elastic connecting wires for electroretinogram (ERG) recording (Figure 2a) [38]. Wang et al. designed a deformable filamentous serpentine carbon belt EP electrode, which can be quickly manufactured by cutting and pasting, with a thickness of only 1.2 µm [39]. The electrode has skin compliance, flexibility, and lightness, similar to spider silk fiber. Then, based on the mathematical concept of Cartan, the substrate free silk serpentine was transferred to a 3D curve skin surface without deformation or wrinkling [39].

The facile fabrication and excellent performance achieved via the geometric engineering preparation method meets requirements for the stretchable EP electrodes mentioned earlier and thus presents a promising strategy for various applications. Geometric engineering is only design at the electrode level and is used as long as it can overcome the mismatch of the mechanical properties of the conductive and substrate materials. However, this method has several problems. First, due to different types of bonding between different materials, geometric engineering may cause delamination. Therefore, it is necessary to design an integrated and inherently stretchable device, which will be introduced later. The goal is to design the microstructure of the conductive material at a specific location so as to prepare a macroscopic stretchable device. The stretching direction of the device depends on the pattern shape, so the final effect of the stretching performance of the device presents anisotropy. The micro-architectural design process often only involves the stretching effect in one dimension (1D), with no tensile deformation in three-dimensional (3D) space. The design of an electrode with excellent stretching effects on a micro pattern requires a more complicated structure [40].

### 2.2. Intrinsic Stretchable Conductor Fabrication

An intrinsically stretchable conductor refers to a material that can maintain a specific electrical conductivity when a certain deformation occurs. Common examples include conductive polymers, liquid metals (LMs), conductive hydrogels, silver nanowires, etc. Compared with those prepared by geometric engineering, electrodes designed based on intrinsically stretchable conductors avoid the use of a multilayer composite process. Therefore, in recent years, research on intrinsically stretchable conductors have yielded many results.

#### 2.2.1. Flexible EP Electrodes Based on Conductive Polymers

A conductive polymer (CP) consists of an insulating base and conductive material in two parts. The matrix is usually a polymer with electrical insulating properties and the “reinforcement” is composed of a number of different forms of conductive charges [43], such as poly(3,4-ethylenedioxythiophene) polystyrene sulfonate (PEDOT:PSS), polypyrrole (PPy), and polyaniline (PANI) [44], etc. Because of their high conductivity, good electrochemical stability [45], and biocompatibility [46], CPs are widely used in EP electrodes. The production process is to change the raw stretchable conductivity of the CP by material processing techniques to obtain excellent charge transportability [36].

CPs can be divided into two types according to their conduction mechanism: filled and intrinsic. Filled CPs are prepared by adding conductive fillers, such as carbon nanoparticles (CNP) or metal powder, to high-molecular polymers with low Young’s modulus. Intrinsic CPs are created by the molecular design of polymers through chemical syntheses, such as optimization through particle doping. The difference between the two types of CPs is that the filled type relies on conductive fillers to obtain conductivity and the polymer itself is not conductive, as it only acts as a binder. The chemical structure of the intrinsic type is changed such that it will undergo a sudden conductivity change of orders of magnitude [47].

Carbon-based conductive polymers are fascinating candidates because of their high specific surface area, good electrical conductivity, and outstanding stretchability. CNTs, carbon fibers (CFs), and their composites with different forms are the most commonly used materials in stretchable EP electrodes [48,49]. Liu et al. mixed CNT as the conductive filler with PDMS to obtain a uniform CNTS-PDMS mixture; the signal amplitude measured by the ECG electrode manufactured using this mixture was equivalent to that of the Ag/AgCl electrode [50].

Cheng et al. presented a soft dry electrode based on polydimethylsiloxane-carbon black (PDMS-CB) CP that was designed and fabricated for continuous, long-term, and stable EOG signal recordings [51]. Their PDMS-CB solution was spin-coated on a glass substrate for 30 s, heated, and dried into a 30 μm film; the film was then transferred to a PDMS substrate through hydrosol tape. The Young’s modulus of this electrode was only 2 MPa and it could be used repeatedly. In their EOG test, the recognition accuracy of eye movements reached 90.63% and performed well in HMI applications [51]. Zhang et al. doped conductive carbon ink composed of 1D CFs and zero-dimension (0D) carbon nanoparticles (CNPs) into PDMS as the conductive filler to prepare a kind of micro-nano hybrid conductive film with EP signal induction [52]. Then, through the die-cutting process they obtained a flexible and dry electrode with serpentine structure design, which could measure the signal activity of ECG, EMG, and EOG [52].

Embedding metal nanoparticles into a stretchable flexible substrate to obtain CPs is also a widely used method. For example, Fan et al. used multi-level Ag nanowires (AgNW) as a conductive filler to uniformly interweave polymer nanofibers and form a large-area elastic conductive film [53]. They prepared a highly stretchable epidermal electrode (SEE) that has a high electrical conductivity of 4800 S/cm. The electrode was still conductive at a high strain of 500% and maintained the initial resistance after 30,000 cycles of tensile strain at 50% or 100,000 cycles of water washing [53].

AgNWs have advantages in the preparation of high-performance flexible transparent conductive electrodes [54,55,56]. Zhou et al. reported a stretchable conductive film prepared by embedding AgNWs just below the surface of a porous thermoplastic polyurethane film by self-assembly process, the obtained film has a transmittance of 61% [57]. However, the biocompatibility of AgNWs is open to discussion [58]. Biocompatibility is an essential factor for the biomedical application of nanomaterials [59], and modification of nanomaterials is usually one of the ways to improve their biocompatibility. Kim et al. introduced CuAuNWs by alkylamine-mediated synthesis in the form of a Au oxidation-resistant layer wrapped around the CuNWs [42]. Then, through an oxidation stability test in a thermal environment, a chemical stability test in sweat, and a biocompatibility test, the sensor was shown to exhibit highly stable performance in the detection of electrophysiological signals, without any oxidation problems (Figure 2f) [42].

Theoretically, more molecular structure of the CP with a π bond represents a greater degree of electron delocalization and the higher the conductivity of the CP [60]. However, it is difficult for the π electrons on the conjugated structure of some CPs to migrate in a long molecular chain when they are not excited. The conductivity will be limited to a certain extent but chemical doping can be used for optimization [61]. For example, Saunieret et al. mixed oxidized CNFs as a dopant with PEDOT and prepared a composite coating on a neural microelectrode array (MEA) by electrochemical deposition [62]. Their experimental results showed that the modified MEA has a specific impedance of 1.28 MΩ μm^2^ at 1 kHz and the charge injection limit is 10.03 mC/cm^2^ [62].

In addition to the abovementioned materials, CPs can also be made of naturally derived biological materials, such as melanin or polydopamine (PDA). However, the use of melanin-like materials is still restricted to biological interface electronic applications [63].

It is well known that an elastomer can maintain its conductivity with the introduction of a small amount of conductive filler and the optimization of the elastomer-filler interface [64]. However, the high loading of conductive fillers affects its mechanical properties, resulting in increased cost. This is similar to how the preparation of intrinsic CP usually faces a trade-off between polymer doping density and stretchability. For example, although carbon nanomaterials perform well, their conductivity dependence on penetration and their poor solubility in most solvents are still obstacles [63,65]. The selection of conductive fillers with excellent conductive properties and suitability for large-scale manufacturing is the focus of current research.

#### 2.2.2. Flexible EP Electrodes Based on LM

LM refers to a metal substance that exhibits liquid fluidity at room temperature. It has both metallic and amorphous properties, good thermal and electrical conductivity, and excellent flexibility. LMs based on gallium (Ga), including pure gallium, eutectic gallium-indium (EGaIn), and GaInSnZn quaternary alloy, can be used as bioelectrode materials because they have good chemical stability and good biocompatibility compared to other LMs, such as Hg and Cs [66].

Due to the excellent fluidity of LM, low-cost and excellent performance PDMS, SEBS, etc., are usually chosen as the base material to prepare LM flexible electronic materials. We broadly divide the combination of LM and substrate materials into two categories: one is similar to the geometric engineering mentioned previously, which uses 3D printing, masking, [67] and laser processing [68] to form surface circuits directly on the substrate; the second type is similar to a CP preparation method, in which LM is injected into microchannels or filled with LM particles/nanoparticles (LMP) [69]. LMPs will serve as conductive path encapsulated in a microfluidic channel [70] or also as nanofiller-level distribution and communication implemented conductive path [71,72,73].

Researchers found the resolution of circuits prepared by LM to not be ideal during experiments and, therefore, not suitable for creating a high-definition device. To solve this problem, LMPs are ideal for this type of preparation method because their small size and large dose significantly reduce the inherent stimulus responsiveness to an internal microenvironment [66]. Dong et al. prepared a highly stretchable electrode array (SEA) using a liquid metal-polymer conductor (MPC) [41]. The SEA was fabricated on a PDMS substrate by screen printing and microfabrication of EGaIn nanoparticles (EGaIn NPs), followed by sputtering deposition of Pt. Experimental results show that the SEA exhibits approximately high stretchability (100%) and excellent cycling stability (>400 cycles) and performs well in biocompatibility experiments (Figure 2b–e) [41]. Marques et al. produced a circuit patch composed of a multilayer embedded flexible printed circuit board based on an EGaIn interconnection [34]. The circuit patch collects EMG signals through the principle of electrostatic coupling. It uses CO_2_ laser ablation of the elastic base material PDMS to fill the LM into the vertical interconnect (VIA) and then connects all the conductive layers. Due to the EGaIn interconnection, the circuit patch can accept a tensile strain of 88.4–95.8%, the corresponding tensile (release) cycle resistance increases (decreases) slightly in the 40% stretched shape, and lasts 1000 times at 0.4 Hz while staying within a reliable range [34]. Ren et al. uniformly dispersed magnetic iron particles in gallium-indium tin LM to form a unique LM-based magnetically active slurry [74]. The mechanical strength of the slurry could be changed by adjusting the size of the magnetic field. The Young’s modulus range could be adjusted from kPa to MPa, and it showed significant advantages in matching the mechanical properties of a skin-electrode interface. However, the application of electrical stimulation of implanted electrodes does not consider the problem of dynamic EP signal electricity supply, in addition to the need for biocompatibility testing [74].

Due to the fluidity, high surface tension, and easy oxidation characteristics of LMs, there are certain challenges to realize its patterning [35]. In addition, the high cost of LM also restricts its applications, and researchers have recently studied the recycling of LM to broaden its scope [75].

#### 2.2.3. Flexible EP Electrodes Based on Conductive Hydrogel

Conductive polymer hydrogel is an important functional material due to its flexibility, similar as that of polymer, and its high conductivity is similar as that of metal [76]. Conductive hydrogel has the advantages of facile synthesis, high biocompatibility, adjustable strength, and versatility. Compared with conventional biosensors, it is more beneficial to integrate conductive hydrogel with electronic device for in vivo EP signal measurement [77,78].

Many EP electrodes are recently developed based on conductive hydrogels since their flexibility and self-adhesion and self-healing properties [79,80]. Liu et al. used Au/PEDOT:PSS hydrogel as a stretchable electrode and simultaneously patterned it with dry etching technology, choosing a dielectric elastomer PFPE-DMA as the packaging material [81]. Their process makes the conductive polymer gel compatible with the traditional photolithography process on a 4-inch wafer, creating an electrode array of fewer than 100 μm in size. In an electromechanical test, the difference in EII between no strain and 20% strain is less than 10%; in the cycle stability test, after 10,000 cycles of 20% tensile strain, the total change in EII is less than 5%. These results showed that the electrode array can withstand the strain generated by a beating heart. Furthermore, the electrode on the heart itself had good adhesion. In order to further enhance the in vivo environment electrode and fit complex cardiac surfaces, the researchers synthesized a bonded electrode array hydrogel auxiliary film comprised of an adhesion layer of bioelectronic bioadhesive (BOBA) polydopamine. In the in vitro test of a pig heart, the electrode array and the low modulus of BOBA minimally affected the normal activity of the heart [81]. Sun et al. used the synergistic effect and hydrogen bond interaction between polyacrylamide (PAM), tannic acid (TA), and PVA to prepare a conductive hydrogel with good adhesion and temperature resistance [82]. The fracture strain of the hydrogel reached 1800% and was successfully applied to ECG and EMG recordings [82]. He et al. prepared a conductive hydrogel fabricated by incorporating tannic acid–carbon nanotubes (TA-CNTs) into a PVA matrix containing a water-glycerol dispersion medium [83]. The TA-CNT-glycerol-PVA (TCGP) hydrogel has excellent anti-freezing performance (at −30 °C) and long-term moisturizing performance (10 days). Making this hydrogel into an electrode could result in the detection of human EP signals in relatively harsh environments [83].

Conductive hydrogels have made rapid progress; their high conductivity and ease of synthesis were realized. However, there are still tremendous opportunities and challenges in their preparation and practical application, especially in realizing multi-functional integration [78,84].

### 2.3. E-Textile Fabrication

A variety of EP signals relevant to clinical analysis were enabled through the preparation EP electrodes within a fabric substrate. [85,86] Researchers mainly prepare fabric EP electrodes according to the different hierarchical structure of e-textiles. First, an external conductive coating is used to make changes directly to the fabric substrate; the other structure is the preparation of conductive fibers or yarns to knit a predetermined pattern to form a conductive fabric [36,87,88,89].

**Figure 3:**
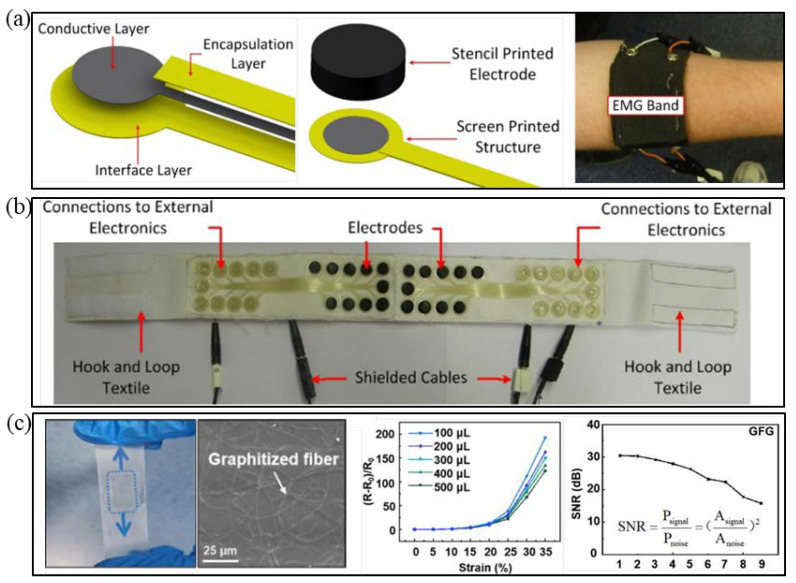
(**a**) Schematic diagram of the details of electrode sites in textile hand band. (**b**) Textile headband for EMG and EOG monitoring. (**a**,**b**) are reproduced with permission from [1]. Copyright 2014, Elsevier. (**c**) Photograph and SEM image (**left**) of the semi-embedded GFG. And resistance change of the semi-embedded GFG as a function of uniaxial stretching strains in middle. And SNR of sEMG detected by GFG/SEBS versus use times (**right**). Reproduced with permission from [26] Copyright 2020, American Chemical Society.

Benefiting from the stretchability, flexibility, and low modulus of elasticity of conductive polymers, [90,91] textile materials, such as cotton or polyacrylonitrile, are easily covered by conductive coatings [86]. Paul et al. used screen printing to print polyurethane paste on fabric as an interface layer (connecting or adhesive layer); afterward, conductive silver paste pathways were coated on the layer and polyurethane paste was used to wrap it and prevent abrasion [1]. Then, a 3-mm-thick conductive rubber electrode contact was pasted at the end of the conductive path as the electrode to directly fit the human skin. Experiments have shown that the device is suitable for detecting EMG and ocular signals, and its performance is comparable to traditional Ag/AgCl electrodes. However, its baseline drift needs to be improved for ECG monitoring (Figure 3a,b) [1]. Gao et al. used the solve thermal method to adsorb a nano-silver structure on cotton fabric [92]. Experiments verified that AgNWs could be effectively attached to cotton through a network structure, increasing the conductivity of the fabric electrode. In a friction test, the square resistance of AgNW/cotton did not increase due to increased friction [92].

Researchers have also produced conductive fabrics based on CP. [93,94,95] However, a CP coating will show durability issues over time that cannot be ignored. For example, Knittel et al. covered a conductive PEDOT layer on textile made of cotton and synthetic fibers by in situ polymerization [96]. Their experiments showed that its electrical properties are unstable with material aging [96]. Homayounfar et al. used the chemical vapor deposition method to prepare reusable hydrogel electrodes on fabric (an eye mask), which could be used continuously for 8 h, and realized wireless eye-tracking through an external circuit to measure EOG signals [97].

Studies have shown that EP electrodes prepared based on carbon-containing e-textile often have higher flexibility and comfort, [98] and their cleanability is more preferable than that of metal-based fabric electrodes [99]. Lam et al. printed multi-walled carbon nanotubes (MWCNT) on soft cotton fabric. Their prepared MWCNT/cotton-based flexible ECG electrode has a simple process and better performance than conventional electrodes [100]. Eskandarian et al. found that the resistance of a silver-based textile electrode increased by 100–300% for 50 washing cycles after washing and testing silver-plated and carbon-containing textile electrodes [101]. Resistance of the carbon-based textile electrode is maintained after about five washing cycles and almost unchanged thereafter [101].

Conductive fibers or yarns can be either based on nonmetallic conductive materials, such as CFs, or metallic materials, such as metal fibers, electrospun fiber mats. Using the conductive thread approach, no additional step is required to establish conductivity after manufacturing the fabric [87]. Qiu et al. reported a transparent graphene skin electrode inspired by the structure of an avian nest [26]. Phenolic resin (PR) was first fixed to a graphene film by electrospinning technology and then the two were more closely adhered by annealing technology. Finally, the graphene film and PR were semi-embedded in SEBS elastomer to make the electrode. Their experimental results showed that the electrode has excellent mechanical and electrical properties, can be used repeatedly, about 10 times, and electrode performance remains unchanged after washing treatment. In the EP signal measurement, the electrode signal-to-noise (SNR) ratio is 30 dB, as shown in Figure 3c [26]. The SNR of 25.6 dB is better than that of traditional Ag/AgCl electrodes [9]. Li et al. reported a highly permeable and stretchable conductor based on an electrospun rubber fiber mat [102]. The conductor exhibited high stretchability, as high as 170%, and the resistance changes only 0.8 under 60% strain. No structural damage or cracks were observed after washing and cyclic stretching. This conductor was successfully utilized as a stretchable yarn and EMG electrode [102].

Current advances in textile technology, new material, nanotechnology, and miniaturized electronics are making wearable system more feasible. Nevertheless, there are still several barriers to functional integration. For example, the process of covering multiple coaxial layers on a fiber substrate still needs to break through in technology and cost. The device performance lifetime, wash ability in the life cycle, and recycling will be great challenges faced by intelligent fabrics in the popularization and application of wearable devices [36]. The requirements for integrating textiles and electronic devices are different at particular levels. For example, conductive yarns require fiber-level manufacturing methods, while combining ordinary fabrics and conductive layers is similar to preparing conductive films. They showed a common target of developing the most effective and high-performance e-textile. Table 1 shows performance comparison the flexible EP electrodes.

## 3. Fabrication Strategies for High-Adhesive EP Electrodes

In the process of long-term dynamic detection of EP signals, if the human body moves or the temperature of the electrode rises during use, the local skin will often perspire. Human sweat will reduce adhesion between the skin and electrode, [109] causing EII elevation, which is not conducive to the measurement of human EP signal. To avoid the influence of sweat and movement, a traditional method is to fix the electrode with tape on the surface of human skin. However, insufficient adhesion of the tape will also cause the abovementioned problems and the use of tape often causes human discomfort. Thus, prepared flexible dry electrodes with adhesion capability is a fundamental strategy to solve the problem of falling due to exercise [21] and a high level of skin adhesion will reduce motion artifact effects.

### 3.1. Intrinsic Adhesive Material Fabrication

The development of material science has made many stretchable substrates with adhesive properties to achieve high adhesion of EP electrodes. Materials such as silk fibroin (SF), [110] polydopamine, and hydrogel [111] are used to prepare stretchable and adhesive polymer materials.

**Figure 4:**
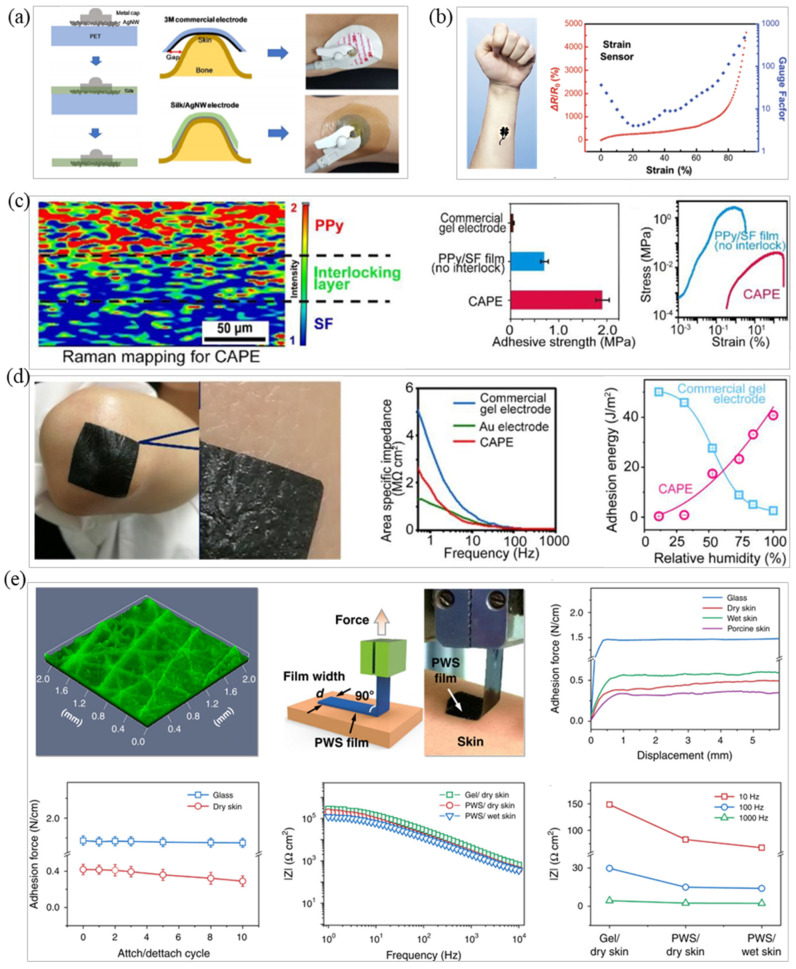
A summary of preparation strategies for high-adhesive electrical physiology electrodes based on intrinsic adhesive material. (**a**) Schematic diagram of the fabrication of a Ag nanowire (AgNW)/silk electrode; schematics and photos of conformal attachment of the AgNW/silk electrode. Reproduced with permission from [113]. Copyright 2018, American Chemical Society. (**b**) Photographs showing a four-leaf clover e-tattoo attached to the forearm and the relative resistance changes as a function of applied tensile strain. Reproduced with permission from [116]. Copyright 2019, John Wiley and Sons. (**c**) Raman mapping for the section of CAPE showing the three-dimensional network structure of PPy and the interlocking structure between PPy and the silk fibroin (SF) gel layer; adhesive strength between the conductive and adhesive layer of CAPE was higher than that of commercial gel electrodes; maximum strain of CAPE was 300%, which was much higher than the 1% for a PPy/SF film fabricated by pasting. (**d**) Highly conformal property of CAPE on a human elbow; CAPE had a lower electrical impedance in the frequency range of 3 to 100 Hz compared with commercial gel and Au/SF electrodes; interfacial adhesion energy of CAPE increased with increasing RH, from 11% to 94.6%, compared with commercial gel electrodes. (**c**,**d**) are reproduced with permission from [109]. Copyright 2020, American Chemical Society. (**e**) Cross-section SEM image of a PWS film conformed on rough skin; setup for the measurement of interfacial adhesion on skin or glass by the standard 90-degree peel test; interfacial adhesion forces of PWS films on glass and various skins; adhesion forces of PWS films on glass and dry skin within ten repetitions of attaching/detaching; impedance spectra of commercial Ag/AgCl gel and PWS dry electrodes on dry and wet skin, and the corresponding impedances at 10, 100, and 1000 Hz. Reproduced with permission from [18]. Copyright 2020, The Authors.

The mechanical properties of SF are regulated by external environmental moisture and coupled with its degradability and biocompatibility. SF is widely used in flexible electronics [112]. Jo et al. combined AgNWs and SF hydrogel to prepare a stretchable electrode with high-adhesion strength and with a tensile strain >400% [113]. After experimental testing, the adhesiveness of the electrode was equivalent to that of the reference adhesive and peel-force measured on the surface of pigskin was 10 N/m (Figure 4a) [113]. Generally speaking, the adhesion of SF is related to its water content. The greater the water content, the higher the bonding strength. SF water content is controlled by the surrounding environment, so further research is needed to prevent the evaporation of SF [114]. Inspired by marine mussels, polydopamine is also often used as an adhesive material for flexible dry electrodes. Xu et al. added dihydroxydopamine (DA) to the polyurethane system in order to obtain improved adhesion of the electrode [115]. They used the dynamic hydrogen bond and dopamine interaction in the system to prepare a kind of elastomer with high viscosity. Then, AgNWs were sprayed on the surface as a conductive layer to prepare a DAE electrode. Experiments have shown that the adhesion strength of the electrode can reach 62 kPa when dry, the tensile deformation is more significant than 100%, and it also has good adhesion in the wet state. The electrode also exhibited good biocompatibility and excellent room temperature self-repairing performance (η ≈ 86%), showing the same signal quality as commercial Ag/AgCl electrodes in an EMG recording [115]. Wang et al. prepared a tattoo electrode based on graphene/silk fibroin/Ca^2+^ (Gr/SF/Ca^2+^), and the electrode has high sensitivity, fast response, and long-term stability [116]. Gr/SF/Ca^2+^ electrodes can maintain stable EII under 50% tensile strain more than 10,000 times and exhibited an excellent SNR in an ECG test for up to 10 h (Figure 4b) [116].

In addition to the extensive research value of conductive hydrogels on intrinsic stretchable conductors, researchers have also studied their adhesion [84]. Lee et al. reacted a gelatin hydrogel mixed with the natural crosslinking agent PEDOT:PSS to obtain an adhesive patch-type hydrogel electrode that can be used in wearable devices to detect EP signals [117]. In their ECG signal detection experiment, the adhesiveness of the electrode patch to human skin was 0.85 N, the patch achieved conformal contact with human skin, and the measured signal quality was comparable with commercial ECG clinical electrodes [117]. Yang et al. used in situ polymerization to prepare a highly adhesive electrode (CAPE) based on conductive hydrogel that is not affected by sweat [109]. CAPE uses Fe ions as the initiator for solution polymerization of SF and PPy monomers to prepare electrodes with stretchability and adhesion. The SF adhesion layer in the electrode decreases the Young’s modulus as the skin–electrode interface water content increases and maintains common contact between electrode and skin, as shown in Figure 4c. Experimental results showed that the electrode can maintain good electrical performance under 30% strain and obtain stable ECG signals during continuous 2-hour dynamic monitoring, Figure 4d shows the comparison of the adhesion between CAPE and commercial gel electrodes. It can be seen from the figure that the adhesion of CAPE is stronger [109].

In addition to the use of SF, polydopamine, hydrogel, etc., researchers have also used other materials to prepare adhesive EP electrodes. For example, Zhang et al. mixed waterborne polyurethane (WPU) and D-sorbitol into PEDOT:PSS to prepare a PWS hybrid film with high conductivity (>390 S/cm) and high stretchability (elongation at break > 43%) [18]. The hybrid film was made into a dry electrode by 3D printing technology, could be closely attached to the skin surface of the human body, and the adhesion was firm; the electrode did not fall off, as shown in Figure 4e. After comparing the working electrode and the reference electrode, the electrode showed excellent performance through clinical testing, including low impedance (82 kΩ cm^2^@10 Hz), high sensitivity, minor motion artifacts, and long continuous working time (no change in 16 h). The signal recording device could detect EEG, ECG, EMG, EOG and other bioelectric signals. The research group used this electrode in a new type of HMI electronic skin, which can control the motion of a robotic arm by detecting EMG signal [18].

High-adhesive electrodes cannot prevent the skin from wicking perspiration and flexible electrodes often face the problem that excessive adhesion may cause damage to the human body when the electrode peels off the skin [118]. Extreme adhesion usually means that the electrode has poor air permeability. A flexible electrode with good air permeability can eliminate the hydration of human skin when the electrode is in everyday contact with skin. However, it is necessary to ensure permeability of the skin and that the electrodes do not lose contact or generate a gap, which will lead to significant reduction in signal amplitude and even fail signal acquisition EP. Therefore, the balance between electrode adhesion and air permeability has gradually attracted the attention of researchers. Thus, adhesion of the prepared electrode is required to be appropriate, reusable, and it is best to be able to adjust the adhesion of the electrode [33]. Table 2 shows performance comparison the adhesive EP electrodes.

### 3.2. Bionic Microstructure Fabrication

**Figure 5:**
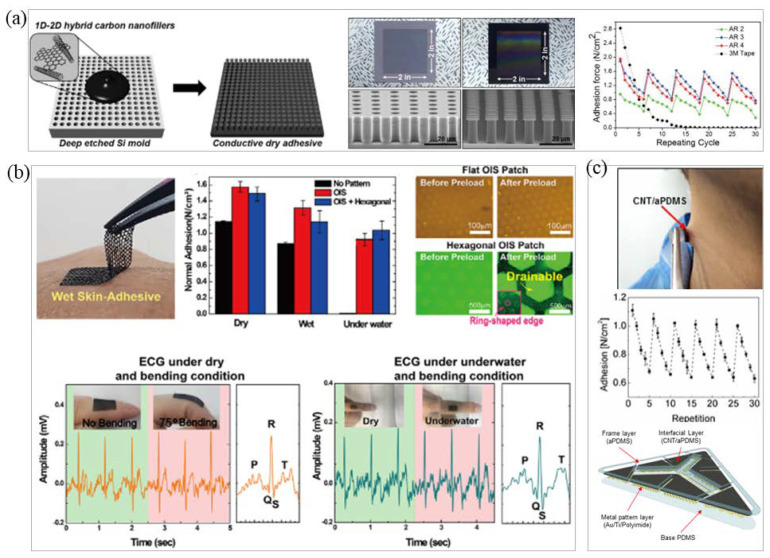
A summary of preparation strategies for high-adhesive electrical physiology electrodes based on bionic microstructures and conductive adhesives. (**a**) Schematic illustration of the fabrication procedure for conductive dry adhesives; digital images of a large-area silicon mold with gecko-inspired micropatterns and a replicated conductive dry adhesive; cyclic adhesion property of conductive dry adhesives compared to a commercial 3 M wet adhesive. Reproduced with permission from [119]. Copyright 2016, American Chemical Society. (**b**) Optical image of a hexagonal mesh-patterned conductive polymer composite (CPC) film with an OIS attached to human skin; normal adhesion strength for flat, OIS, and hexagonal mesh-patterned films with an OIS in dry, wet, and underwater conditions on a skin replica with a preload of 1.0 N cm^−2^; fluorescence microscopic images representing conformal contact of a HMP OIS patch underwater; biosignals (ECG) detected by CPC stretchable electrodes attached to a human finger with 0 and 75° bending and in underwater conditions. Reproduced with permission from [28]. Copyright 2020, American Chemical Society (**c**) Adhesiveness of CNT/aPDMS, adhesion force measurements during repeated attaching and detaching processes, and structure of an ECG. Reproduced with permission from [29]. Copyright 2014, The Authors.

In order to achieve the repeatable adhesion of EP electrodes, scientists were first inspired by biology and discovered that materials with bionic micropillars or sucker-like structures, [120] such as those observed in geckos [121] and octopuses, can be stretched and adhered [18]. For example, due to the layered structure and a large number of micro-nano pillars on gecko antennae, geckos can repeatedly adhere to rough surfaces. Kim et al. prepared a conductive dry adhesive (CDA) based on this principle and combined it with elastic carbon nano composite materials [119]. The prepared CDA pad showed high normal adhesion force (~1.3 N/cm^2^), even on rough human skin, and excellent cyclic performance for repeatable use, over 30 times, without degradation of adhesion force. The body-attachable CDA can be used for measuring biosignals under daily conditions (e.g., underwater, with movements) due to its superior conformality and water repellent characteristic, as shown in Figure 5a [119]. Inspired by the structure of the octopus sucker, Min et al. prepared a stretchable conductive polymer composite (CPC) patch with a hexagonal grid structure that can achieve water and air permeability [28]. The EP signal can be stably measured by creating an ECG electrode and the adhesion can also be maintained under water (Figure 5b) [28]. Liu et al. combined intrinsic SF with biomimetic microcolumn to prepare an MSFA film with adjustable adhesion strength using the spin coating method to achieve permeability and easy stripping of a high-adhesion electrode [122]. The adhesion was mainly controlled by the diameter and density of the microcolumn. The MSFA film had excellent easy stripping and reusability [122].

It is worth noting that the adhesion of bionic microstructure to the skin is easily affected by sweat or dirt secreted on the skin and accumulation or pollution of the structure. In addition, the adhesion of these structures can also bring discomfort to patients with skin injuries [18].

### 3.3. Conductive Adhesive Fabrication

Conductive adhesives can overcome the problem of inadequate electrode adhesion. In fact, requirements for conductive adhesives are to maintain high-strength adhesion-not only to be used in daily environments but also in special environments (e.g., underwater, extreme temperature) to ensure that the electrode does not fall off the human skin or tissue and to measure continuous and stable EP signal.

In conductive adhesive research, there are many studies on the use of carbon and composite materials to prepare adhesive and resistive electrodes [123]. Lee et al. used a CNT/PDMS layer to cover a metal-patterned polyamide (PI) electrode to make a stickable CNT electrode [29]. Due to the softness and adhesion of the CNT/PDMS layer, it could penetrate wrinkles of the epidermis and maintain firm contact (Figure 5c) [29]. Inoue et al. prepared a polymer adhesive based on hydrophilic polyurethane (PU) [124]. The thickness of the adhesive layer was only a few nanometers, which was sufficient to form an interpenetrating polymer network with a CP to realize the adhesion of the flexible base and interface material. After testing, the shear strength between the interface increased by three orders of magnitude, exceeding 120 kPa. This method is compatible with various manufacturing methods for conductive polymers without compromising electrical or mechanical properties [124]. Yuk et al. used biopolymers (gelatin or chitosan) and crosslinked polyacrylic acid grafted with N-hydrosuccinimide ester to prepare dry double-sided tape (DST) [125]. The adhesion mechanism is first the removal of interfacial water from the surface of human skin or tissue, then a temporary crosslinking reaction occurs and covalent crosslinking with the amine group on the surface of the skin or tissue further improves the adhesion stability and strength of the DST. Experimental tests showed that the adhesive will form a strong adhesion with a bonding strength greater than 120 kPa and an interface toughness of more than 710 J/m^2^ within 5 s when in contact with wet pigskin; the adhesive strength remained unchanged after 14 days [125]. Tian et al. proposed a method to fabricate a large-area adhesive biosensor [126]. PI and Cr/Au were used to form a predesigned electrode pattern on a silicon wafer by etching, deposition, and photolithography. Then, water-soluble adhesive tape (3 M) was transferred to the prepared adhesive silicone carrier to complete the encapsulation and fabrication of the electrode. The carrier of the electrode is composed of two layers of organic silicon. The skin contact layer contains organic silicone adhesive, which can form a conformal contact with the skin, has no irritating adhesive interface, and has excellent air permeability due to its microperforated structure. Experiments showed that this method can prepare a large-area electrode of 17 cm × 13 cm with a stretch rate of 18% and a water vapor transmission rate greater than 85%. High-resolution EEG, EMG, and EOG signals were collected in bioelectrical signal testing [126]. Sun et al. prepared a non-swelling protein adhesive with solid adhesive properties; this material relies on electrostatic complexation between highly charged polypeptides (SUPs) and oppositely charged biomimetic synthetic surfactants to form a super viscous coacervate [127]. Experiments showed that it exhibits durable adhesive properties on human tissues [127]. Dou et al. prepared a stretchable conductive adhesive for connecting rigid electronic devices and stretchable circuits. The conductive adhesive is a metal-polymer-based adhesive that uses PDMS as a flexible substrate and LM and CNT together to form a conductive network [128]. Experimental results showed that the tensile strain of the conductive adhesive is about 40% and the conductivity remains stable when deformed [128].

In recent years, tattoo-like skin sensors have become an emerging research topic due to their thin and soft characteristics [21,129]. Ameri et al. designed a graphene-based tattoo electrode (GET) with a total thickness of 463 ± 30 nm, a transparency of 85%, and a stretchability of more than 40% [27]. It can be directly laminated on the human skin, like a temporary tattoo, and kept on the skin for several hours without delamination. Experimental tests showed that the EII of the GET can be on par with traditional Ag/AgCl electrode while providing excellent comfort, mobility, and reliability. GETs are successfully used to measure ECG, EMG, and EEG [27]. The emergence of air-permeable, transparent and ultra-thin electrodes has enriched the additional functions of highly adhesive flexible EP electrodes, which also means that the future development of adhesive electrodes will be closer to human life.

## 4. Processing Technology for High-Adhesion Flexible EP Electrodes

Modern manufacturing can be divided into two main categories: subtractive and additive [130]. Most manufacturing processes use subtractive patterning methods and the deposition of materials under high temperatures and ultra-vacuum conditions, with all its disadvantages [131]. On the contrary, coating, printing, 3D printing, and other processes based on additive manufacturing are gradually being applied to the preparation of flexible EP electrodes and have gradually shown its advantages in the flexible EP electrode manufacturing market.

### 4.1. Manufacture of Micro-Nano Processing Technology

The development of a flexible EP electrode preparation method based on micro-nano processing technology is relatively mature and mainly involves the micro-nano patterning process and thin film deposition technology. Its actual operation mode is relatively fixed. For example, the patterning of flexible substrates is usually realized by means of laser engraving [106,130] and ion etching, while the adhesion of conductive film on the substrate is mainly realized by vapor deposition technology, such as evaporation, sputtering, [132] and chemical vapor deposition (CVD) [133]. Jin et al. first used photolithography to engrave a predesigned template pattern on polymethylmethacrylate (PMMA) and then casted a Ag/PDMS mixture on PMMA to successfully fabricate a flexible ciliated electrode, which required a certain amount of pressure during ECG signal testing [134]. Jung et al. formed electrode patterns on a flexible substrate PDMS by photolithography and then used magnetron sputtering technology to deposit a Ti/Ag conductive layer on the PDMS to prepare an sEMG electrode [135]. Fiedler et al. used multiphase DC magnetron sputtering technology to deposit a thin layer of conductive titanium nitride on a polyurethane substrate and carried out a patterned design that fits the skin [136]. In the end, the same signal quality as the traditional Ag/AgCl electrode was obtained [136].

In order to manufacture flexible EP electrodes with better performance or to enhance specific properties of the electrodes, the preparation process can be improved. As mentioned earlier, the GET uses a “wet transfer, dry patterning” manufacturing process. “Wet transfer” refers to a step of copper etching that retains high continuity of large-area graphene grown on copper foil. “Dry patterning” refers to use of a programmable mechanical plotter to engrave the designed filamentous serpentine shape on graphene. Compared with photolithography, dry patterning can minimize the chemical pollution of graphene and significantly improve the time and cost-effectiveness. Graphene is firstly grown on metal foil by CVD, a flexible substrate is then spin-coated on the foil, and the metal foil is peeled off to a paper substrate that can be carved into a fixed pattern by a mechanical knife to complete the patterning process, finally removing surplus [27]. Yun et al. used nanoscale gold particles to modify the surface of traditional metal electrodes and optimized the parameter settings of the nano-deposition process to increase the SNR of the electrode by increasing the specific surface area. The experimental results showed that the SNR increases by 51% in an ECG test and 63% in an EMG test [137]. Finally, through the quantitative detection of motion artifacts, it was found that the motion noise of the electrode was reduced by 95% compared with a traditional Ag/AgCl electrode [137].

In additive manufacturing, the material processing method becomes a single layer stacking process. The required materials (e.g., direct transfer, embedding, embossing, etc.) are printed directly and selectively on top of the substrate. Compared with material reduction technology, waste is avoided [138]. These advantageous features of printed electronics are highly promising for future high-definition printed sensing system, low-power consumer electronics, and large-scale integration of electronics [139].

### 4.2. Manufacture of Printed Electronic Technology

The aforementioned micro-nano processing-based lithography, ion etching, CVD, and other processes have high costs, low throughput, and require micro-processing in a traditional clean room [30]. Printed electronics (PEs) technology has taken an important step towards the design of next generation of bioelectronic interface with rapid and simple prototyping methods [33,130,140,141].

#### 4.2.1. Manufacturing Based on 2D PEs

The coating process is a simple processing method that is often used when preparing EP electrodes based on an e-textile fabrication strategy [86]. Yapici et al. put nylon fabric into a diluted graphene oxide (GO) suspension and then chemically converted GO into rGO to prepare graphene-coated textile ECG electrodes [142]. Their experimental test showed that the EII and ECG signal quality of the electrode have a 97% correlation with a traditional Ag/AgCl wet electrode [142]. Jiang et al. prepared a flexible electrode based on Ag flake/PDMS electrically conductive composite (ECC) for multiple EP signal detection [143]. The electrode uses the bar coating method to coat the ECC material on the substrate and other components. The electrode exhibited a higher SNR ratio in the EP signal measurement experiment than the traditional Ag/AgCl electrode during ECG measurement. The sensitivity to motion artifacts was low and the electrode also showed good signal quality in EEG and EMG signal measurement [143].

**Figure 6:**
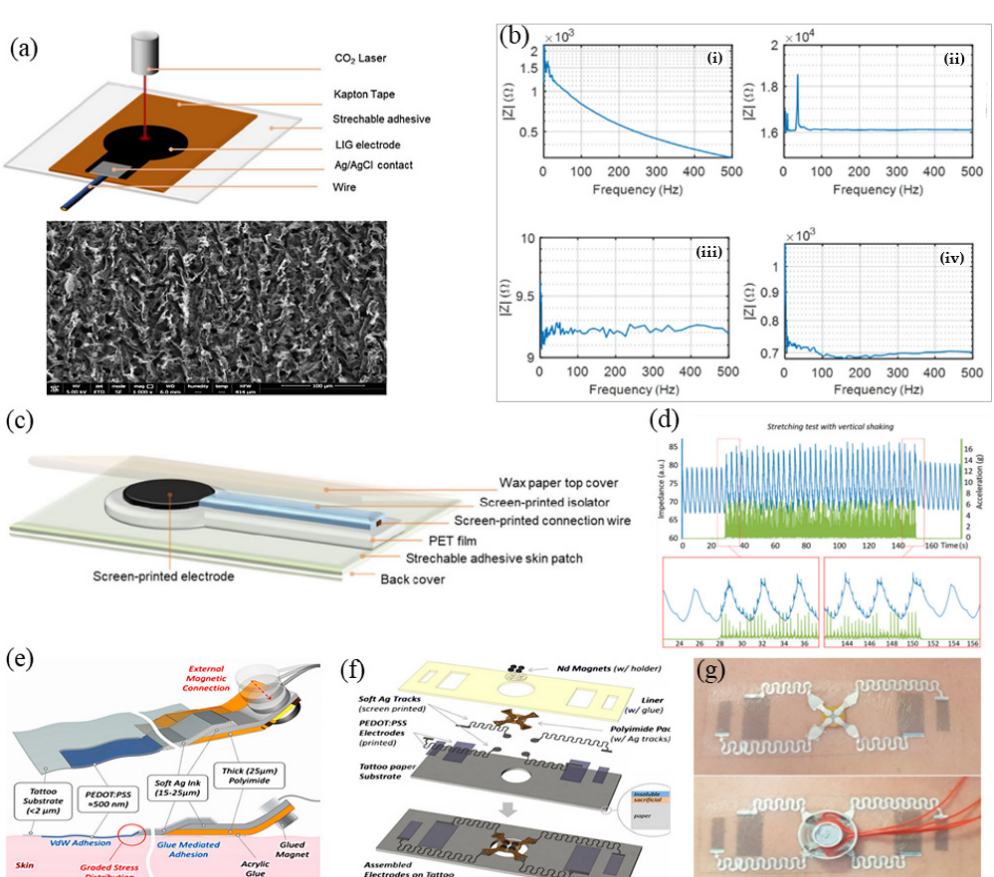
Partial summary of electrical physiology electrode preparation strategies based on printed electronics technology. (**a**) LIG electrode and its SEM. (**b**) Impedance measurement for (**i**) commercial electrodes, (**ii**) carbon electrodes, (**iii**) silver electrodes and (**iv**) LIG electrode. (**c**) Multilayer structure used for each electrode from screen printed electrodes. (**a**–**c**) are reproduced with permission from [6]. Copyright 2020, The Authors. (**d**) Example of impedance signal during 2 min of “shaking”, showing that the sensor output is scarcely affected by motion artifacts. (**e**) Connection schematic section for TTE electrode. (**f**) Schematic representation of design and geometry of PEDOT:PSS electrodes and interconnections for transthoracic impedance measurements. (**g**) TTE electrode released on the chest of a subject (top) and the same docked through a magnetic connector used for signal acquisition. (**d**–**g**) are reproduced with permission from [103]. Copyright 2021, The Authors.

Screen and inkjet printing are key tools widely applied in PEs that need to be addressed for use with conductive materials. LM is most suitable as a conductive ink to prepare EP electrodes by the printing process because of its inherent fluidity and high conductivity. Current technology can achieve good control to a certain extent. Guo et al. proposed a “one-step liquid metal transfer printing” method, which uses the chemical interaction between the adhesive polymer and LM to overcome the mechanical mismatch between the flexible substrate and the interface material by printing [144]. Even on substrates with weak wettability to LM, their method can produce complex conductive geometries well [144]. Tang et al., through the screen-printing process, used the geometric engineering preparation method by first embedding patterned LMP into the polymer through flow and stripping steps to generate the surface metal-polymer conductor (MPC) [145]. Because MPC has the advantages of printing and mass copying, EP electrodes based on MPC are not only tensile and have high conductivity, but also greatly reduce production cost [145]. Zeng et al. manufactured an EMG electrode by hydrographic printing a silver-indium-gallium alloy system on tattoo paper [146]. After the alloy system solidified, the adhesive sheet and the support sheet were bonded through a glue layer. Afterward, the bonding system was soaked in water and the electrode made of the alloy drops off; after drying the electrode can be used. Experiments showed that the impedance of their electrodes is less than that of a commercial electrode and exhibited good performance in abrasion resistance [146]. Shur et al. prepared a conductive hydrogel using a coating formula of mixed polyacrylamide and PEDOT:PSS. They prepared a soft electrode on PDMS by screen printing [147]. The layer solves the matching problem between the neural interface device and the human tissue, has an adjustable elastic modulus in the range of 10–100 kPa, and its electrochemical properties are equivalent to hard conductive ink, making it useful for the production of flexible nerve microelectrodes [147]. Toral et al. used innovative materials, such as laser-induced graphene (LIG) and Pes. Three types of electrodes based on LIG, silver chloride, and carbon inks, are compared during the acquisition of bipotentials, such as ECG, EMG, and EOG [6]. In their measurement of electrode impedance, LIG and commercial electrodes tend to have a capacitive behavior while screen printed electrodes have purely resistive behavior. The silver electrode has the lower impedance while the highest impedance was observed for that of carbon. Commercial and LIG electrodes exhibited the similar impedance. In terms of noise, carbon and AgCl electrodes capture more noise than those of commercial and LIG electrodes. In the case of ECG acquisition, all the tested systems were able to acquire ECG without distortion. In addition, by comparing the production costs of the three electrodes, it was found that the production cost of the commercial electrode is the lowest, followed by the LIG and carbon electrodes, and the AgCl dry electrode had the highest cost (Figure 6a–c) [6]. Bihar et al. used inkjet printing of PEDOT:PSS on environmentally friendly and recyclable commercial paper to prepare ECG electrodes, which can be used for ECG measurement of finger contact [148]. Taccola et al. used inkjet printing technology to print PEDOT:PSS on temporary tattoo paper and prepare a dry and imperceptible temporary tattoo electrode (TTF) [103]. Through the docking of TTF and external equipment, the EP signal of a human body could be detected, and its performance met experimental verification under stretching (up to 10%) and changing with time (up to 96 h) (Figure 6d–g) [103]. Ferrari et al. easily prepared an ultra-thin electrode tattoo array by inkjet printing CP onto commercial decal transfer paper, the thickness of which was less than 1 µm [149]. Compared with the traditional Ag/AgCl wet electrode, the feasibility of application of their electrode array to wearable devices was verified in an EP signal test of ECG and EMG [149]. Lo et al. reported a stretchable polymer blend prepared by mixing PEDOT:PSS with polar solution additives, such as ethylene glycol [150]. The blend could be patterned by an inkjet printing process and at the same time exhibits lower sheet resistance (58 Ω/sq) and greater mechanical deformability. In their experiment, the polymer blend was printed on PDMS to prepare a flexible electrode that could be used for ECG detection [150].

Screen and inkjet printing are key tools widely applied in PEs that need to be addressed for use with conductive materials. LM is most suitable as a conductive ink to prepare EP electrodes by the printing process because of its inherent fluidity and high conductivity. Current technology can achieve good control to a certain extent. Guo et al. proposed a “one-step liquid metal transfer printing” method, which uses the chemical interaction between the adhesive polymer and LM to overcome the mechanical mismatch between the flexible substrate and the interface material by printing [144]. Even on substrates with weak wettability to LM, their method can produce complex conductive geometries well [144]. Tang et al., through the screen-printing process, used the geometric engineering preparation method by first embedding patterned LMP into the polymer through flow and stripping steps to generate the surface metal-polymer conductor (MPC) [145]. Because MPC has the advantages of printing and mass copying, EP electrodes based on MPC are not only tensile and have high conductivity, but also greatly reduce production cost [145]. Zeng et al. manufactured an EMG electrode by hydrographic printing a silver-indium-gallium alloy system on tattoo paper [146]. After the alloy system solidified, the adhesive sheet and the support sheet were bonded through a glue layer. Afterward, the bonding system was soaked in water and the electrode made of the alloy drops off; after drying the electrode can be used. Experiments showed that the impedance of their electrodes is less than that of a commercial electrode and exhibited good performance in abrasion resistance [146]. Shur et al. prepared a conductive hydrogel using a coating formula of mixed polyacrylamide and PEDOT:PSS. They prepared a soft electrode on PDMS by screen printing [147]. The layer solves the matching problem between the neural interface device and the human tissue, has an adjustable elastic modulus in the range of 10–100 kPa, and its electrochemical properties are equivalent to hard conductive ink, making it useful for the production of flexible nerve microelectrodes [147]. Toral et al. used innovative materials, such as laser-induced graphene (LIG) and Pes. Three types of electrodes based on LIG, silver chloride, and carbon inks, are compared during the acquisition of bipotentials, such as ECG, EMG, and EOG [6]. In their measurement of electrode impedance, LIG and commercial electrodes tend to have a capacitive behavior while screen printed electrodes have purely resistive behavior. The silver electrode has the lower impedance while the highest impedance was observed for that of carbon. Commercial and LIG electrodes exhibited the similar impedance. In terms of noise, carbon and AgCl electrodes capture more noise than those of commercial and LIG electrodes. In the case of ECG acquisition, all the tested systems were able to acquire ECG without distortion. In addition, by comparing the production costs of the three electrodes, it was found that the production cost of the commercial electrode is the lowest, followed by the LIG and carbon electrodes, and the AgCl dry electrode had the highest cost (Figure 6a–c) [6]. Bihar et al. used inkjet printing of PEDOT:PSS on environmentally friendly and recyclable commercial paper to prepare ECG electrodes, which can be used for ECG measurement of finger contact [148]. Taccola et al. used inkjet printing technology to print PEDOT:PSS on temporary tattoo paper and prepare a dry and imperceptible temporary tattoo electrode (TTF) [103]. Through the docking of TTF and external equipment, the EP signal of a human body could be detected, and its performance met experimental verification under stretching (up to 10%) and changing with time (up to 96 h) (Figure 6d–g) [103]. Ferrari et al. easily prepared an ultra-thin electrode tattoo array by inkjet printing CP onto commercial decal transfer paper, the thickness of which was less than 1 µm. Compared with the traditional Ag/AgCl wet electrode, the feasibility of application of their electrode array to wearable devices was verified in an EP signal test of ECG and EMG [149]. Lo et al. reported a stretchable polymer blend prepared by mixing PEDOT:PSS with polar solution additives, such as ethylene glycol. The blend could be patterned using an inkjet printing process and at the same time exhibits lower sheet resistance (58 Ω/□) and greater mechanical deformability. In their experiment, the polymer blend was printed on PDMS to prepare a flexible electrode that could be used for ECG detection [150].

**Figure 7:**
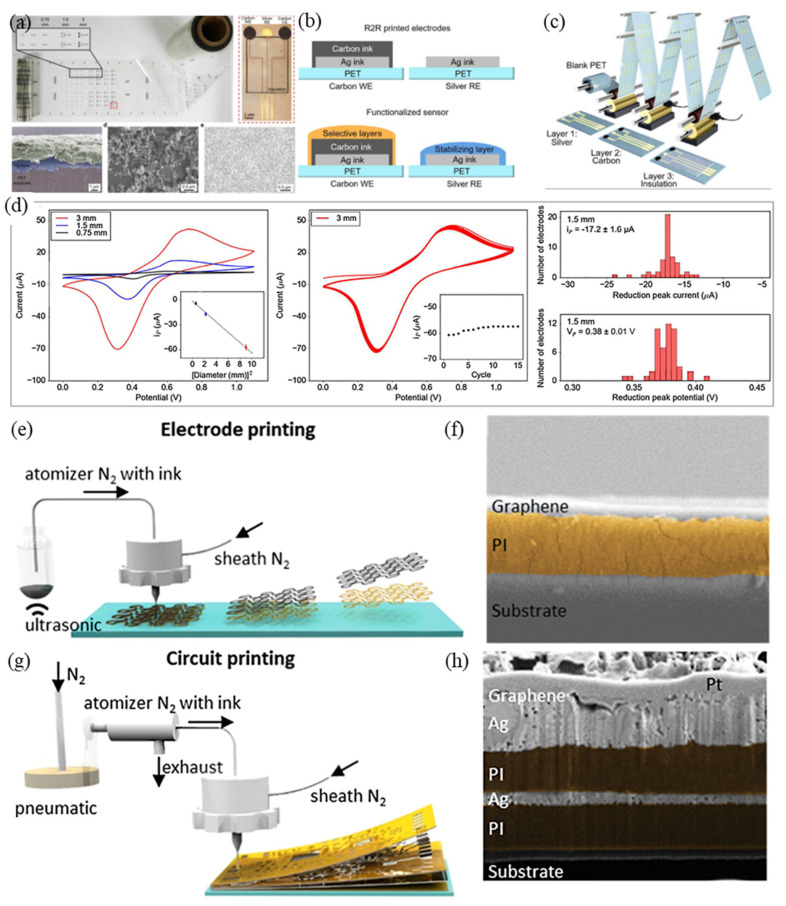
Partial summary of electrical physiology electrode preparation strategies based on printed electronics technology. (**a**) Photograph of a roll-to-roll gravure printing biocompatible electrode array. (**b**) Cross-section schematics of gravure printed carbon and silver electrodes after printing and then after functionalization. (**c**) Roll-to-roll gravure printed electrodes on a 150-m roll of PET substrate. (**d**) Cyclic voltammogram (CV) of the gravure printed electrode; the multi-cycle CV of the 3-mm electrode array shows good stability; restore peak current and its transformation. (**a**–**d**) are reproduced with permission from [20]. Copyright 2018, American Chemical Society. (**e**) Illustration of the printing of nanomaterials to fabricate a nanomembrane electrode. (**f**) Cross-sectional scanning electron microscopy (SEM) image showing a multilayered sensor structure. (**g**) Illustration of the printing of nanomaterials to fabricate circuit interconnects. (**h**) Focused ion beam (FIB)-assisted SEM image (**f**–**h**) are reproduced with permission from [30]. Copyright 2020, American Chemical Society.

Fabric electrodes based on conductive polymers can also be developed using conductive inks through screen printing or inkjet technology. Inkjet printing can be used to produce electroactive textile materials in a one-step agglomeration process of a conductive layer on a substrate [86]. Gao et al. used inkjet printing to directly print Ag films on textiles for wearable electronic devices [92]. In addition, other printing methods have gradually been applied in the preparation of flexible EP electrodes. Liu et al. used AgNW as a layer to print on PDMS to prepare a self-healing, solid, and stretchable electrode [151]. Their experimental results showed that the electrode has friction resistance and is self-healing, which provided a new idea for the preparation of self-healing electrodes based on printing technology [151]. Bariya et al. proposed a method for preparing R2R gravure printing electrodes, which realized a device with uniform redox kinetics for printing on a 150-m-long flexible substrate roll (Figure 7a–d) [20]. Kwon et al. developed a graphene electromyographic electrode prepared by air spray printing (AJP) process [30]. PI ink was sprayed on a PMMA/glass substrate [30]. Then, the graphene ink was sprayed on the PMMA/glass substrate after thermal curing. After drying, the sacrificial layer of PMMA was dissolved by acetone solution and the electrode was transferred to the prepared elastic film by water-soluble adhesive tape. Their results showed that the resistance change of the electrode after 60% stretching deformation lasting 500 times was negligible. In a human–computer interaction experiment of controlling a mechanical arm, the frontal myoelectric signal obtained by this electrode could classify six types of muscle activity, with an accuracy rate of over 97% (Figure 7e–h) [30].

#### 4.2.2. Manufacturing Based on 3D PEs

**Figure 8:**
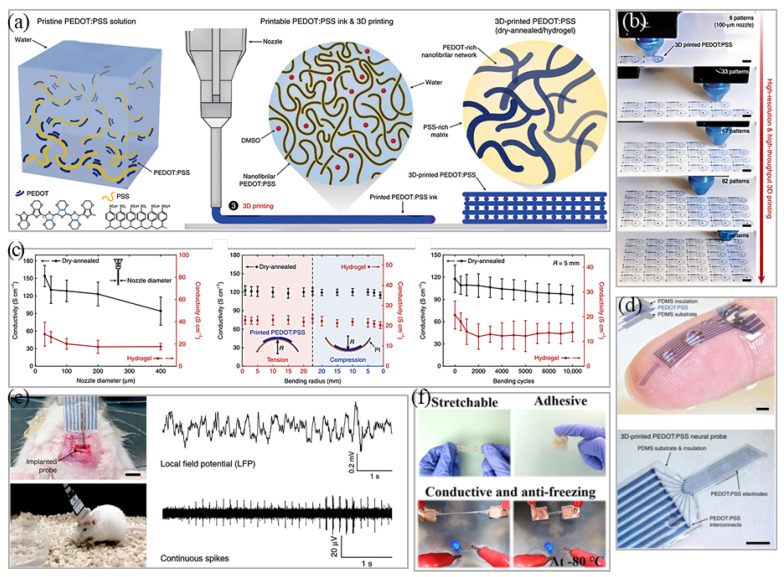
A summary of preparation strategies for electrical physiology electrodes based on 3D printing. (**a**) Design of 3D printable conducting polymer ink. (**b**) Sequential snapshots for 3D printing of high-density flexible electronic circuit patterns by the conducting polymer ink. (**c**) Conductivity as a function of nozzle diameter for 3D-printed conducting polymers in dry and hydrogel states; conductivity as a function of bending radius; conductivity as a function of bending cycles. (**d**) Image of the 3D-printed soft neural probe with nine channels by the conducting polymer ink and the PDMS ink. (**e**) Images of the implanted 3D-printed soft neural probe (left) and a freely moving mouse with the implanted probe; representative electrophysiological recordings in the mouse dHPC by the 3D-printed soft neural probe (right). (**a**–**e**) are reproduced with permission from [106]. Copyright 2020, The Authors. (**f**) Conductive hydrogel performance based on 3D-printing technology in extreme environments. Reproduced with permission from [154]. Copyright 2021, Elsevier.

3D printing is also known as additive manufacturing (AM) technology. The progress of AM has opened up new prospects for next generation design and manufacturing of composite materials. Composite materials carry out spatial digitization and physical layout of materials/structures in point-to-point printing [152]. Zhang et al. used a mixed solution of 2D GO dispersed in sodium alginate viscous solution as 3D printing ink and then fixed it on a PET film at a controllable temperature for wire drawing [153]. After drying, the film was immersed in 0.5 M CaCl_2_ aqueous solution for 5 s and then transferred to deionized water to swell into spring. The maximum tensile strain of the printed ribbon was more than 1000%. The ribbon was wound around a bullfrog sciatic nerve and successfully recorded nerve signals [153]. Yuk et al. introduced a high-performance 3D printable CP ink based on PEDOT:PSS that could easily produce high-resolution (more than 30 µm) and high aspect ratio (more than 20 layers) microstructure [106]. Their 3D-printed CP conductivity exceeded 155 S/cm and could be transformed into high conductivity (conductivity 28 S/cm) and soft (Young’s modulus < 1.1 MPa) hydrogel microstructure in a wet environment (Figure 8a–e) [106]. Chen et al. produced a kind of conductive hydrogel and adhesive based on 3D printing technology. The hydrogel was characterized by an adhesive electrode prepared under an extremely low temperature (−80 °C) [154]. It could also accurately collect ECG, EMG, and EOG signals. In a tensile test, the elongation of conductive hydrogel was up to 3920% and the adhesion strength reached 61 kPa. Researchers applied the hydrogel to HCI and it showed excellent performance (Figure 8f) [154].

Although PEs open up a new class of potential applications that is beyond the reach of traditional microelectronics, 2D printing accuracy and layer-by-layer overprinting accuracy of each layer of material in the AM process are the keys to electrode electrical performance. In addition, the AM of functional devices generally involves more than two kinds of materials; there are different AM technologies to choose depending on the materials used, their functionality, and their rheology. Therefore, the matching between the integration of equipment and the printing process is also one of the challenges faced by researchers.

## 5. Conclusions

With the rapid development of new flexible electronic materials and devices, researchers have put forward new ideas regarding the stretchability, adhesion, and batch preparation of EP electrodes. These ideas have provided a guarantee for accurate and stable human bioelectrical signal collection and also afford new opportunity for the development of a wearable human–computer interaction system.

Compared with traditional electrodes, high-adhesion flexible EP electrodes have greatly improved performance. However, these electrodes still need to be optimized in terms of electrode stability, response speed, and reliability of clinical testing. The following challenges are still being faced, especially for future smart applications as follows. (1) Tensile stability and charge transfer efficiency of flexible EP electrodes are too sensitive to the properties of the material itself, therefore, facing insufficient adaptability under different multiple environments. (2) The use of adhesive EP electrodes in practical applications is often affected by the differences in the skin of individual organisms and the environment in which they are used. In order to improve the comfort and safety of users, it is necessary to detect differences in the adhesive strength of electrodes with different types of EP signals; chemical research needs to be discussed. (3) A low-cost batch preparation process is one of the foundations for the industrialization of high-viscosity flexible EP electrodes. However, the cost of preparation technology based on printed electronic processes also increases with the increase in electrode accuracy. Optimization and reform of the preparation process can be achieved and high-viscosity flexible EP electrodes with better performance pave the way. We believe that in the future, high-adhesion flexible EP electrodes will be integrated with more new technologies and materials. It is only a matter of time before they are widely used in smart human–computer interaction.

## Figures and Tables

**Table 1:** Performance comparison of flexible EP electrodes.

Structures	EP Signal Type	Conductivity/Resistivity	SNR/RMS	Stretchability	Thickness	Cycling Stability	Ref.
AgSEBS/disposable soft contact lens	EOG	18.2 ± 3.8 Ω	/	350%	70 μm	1500	[38]
PEDOT:PSS/Ag/PI	EMG&ECG	ΔR/R = 0.1–1 Ω @64 kHz (under 10% strain)	SNR ≈ 20.7 dB vs. SNR ≈ 22.4 dB (Conv. ^1^)	110%	≈1.7 μm	/	[103]
PDMS/EGaIn NPs	ECG	ΔR/R < 7% (under 10% strain)	/	≈100%	100 μm	>400	[41]
PDMS/AgNW	ECG	≈35 Ω/sq	RMS ≈ 500 µV vs. RMS ≈ 450 µV (Conv.)	≈400%	≈2 mm	1000	[104]
PEDOT: PSS/WPU/ D-sorbitol	ECG&EOG&EEG	545 S/cm	RMS ≈ 25 μV vs. RMS ≈ 28 μV (Conv.)	130%	20 μm	40	[18]
PEDOT:PSS/LIG	ECG	17.4 Ω/sq@10 Hz	SNR ≈ 12.9 dB vs. SNR ≈ 13.3 dB (Conv.)	/	100 μm	/	[105]
PEDOT:PSS ink	/	155 S/cm	/	113%	100 μm	/	[106]
GFG/SEBS	EMG&ECG&EEG	≈150 Ω/aq	SNR ≈ 30 dB	35%	/	10	[26]
SBS/Ag-AuNWs	ECG&EMG	72,600 S/cm	/	≈840%	≈20 µm	3000	[107]
SF/Au	EMG	7 Ω/sq	SNR ≈ 17.17 dB vs. SNR ≈ 17.95 dB (Conv.)	>400%	160 µm	7500	[108]

^1^ Conventional commercial Ag/AgCl electrodes.

**Table 2:** Performance comparison of adhesive EP electrodes.

Materials	EP Signal Type	Adhesion	Other Performance	Ref.
TA-PVA-PAM organohydrogel	ECG	80 kPa	UV-blocking > 90%	[82]
PDMS/dopamine/AgNWs	EMG	~62 kPa	≈16 kPa (underwater)	[115]
PU/CNT/Ag	ECG	~1.5 N/cm^2^	cycling stability ≈ 1000	[28]
GET electrode	ECG&EMG&EEG	/	Transparency ≈ 85%	[27]
PDMS/graphene	ECG	~1.3 N/cm^2^	volume resistance (~100 Ohm·cm)	[119]
